# Influence of Mirror Therapy (Specular Face Software) on Electromyographic Behavior of the Facial Muscles for Facial Palsy

**DOI:** 10.3390/brainsci11070930

**Published:** 2021-07-14

**Authors:** Alfonso Gil-Martínez, Sergio Lerma-Lara, Alfredo Hernando-Jorge, Ana Campos-Vegas, Audrey Aceval, Rafael Pagés-Scasso, Francisco Morán-Burgos, Hector Beltran-Alacreu

**Affiliations:** 1 Centro Superior de Estudios Universitarios La Salle, Department of Physiotherapy, Universidad Autónoma de Madrid, 28023 Madrid, Spain; sergio.lerma@lasallecampus.es (S.L.-L.); aherjo@campuslasalle.es (A.H.-J.); acamve@campuslasalle.es (A.C.-V.); aace@campuslasalle.es (A.A.); 2 CranioSPain Research Group, Centro Superior de Estudios Universitarios La Salle, 28023 Madrid, Spain; hector.beltran@uclm.es; 3 Unit of Physiotherapy, Hospital La Paz Institute for Health Research, IdiPAZ, 28046 Madrid, Spain; 4 Motion in Brains, Centro Superior de Estudios Universitarios La Salle, 28023 Madrid, Spain; 5 Volograms Limited, D08FN36 Dublin, Ireland; rafa@volograms.com; 6 Grupo de Tratamiento de Imágenes (GTI), Escuela Técnica Superior de Ingenieros de Telecomunicación, Universidad Politécnica de Madrid, 28040 Madrid, Spain; fmb@gti.ssr.upm.es; 7 Toledo Physiotherapy Research Group (GIFTO), Faculty of Physical Therapy and Nursing, Universidad de Castilla-La Mancha, 45071 Toledo, Spain

**Keywords:** biofeedback, facial paralysis, mirror therapy, software, electromyography, motor control

## Abstract

*Introduction:* Facial paralysis (FP) is a neuromuscular disorder caused by facial nerve injury. There are two main types of FP (which can be either primary or secondary): central and peripheral; *Procedure of cases:* This case series presents five patients with facial paralysis with different etiologies. In all cases, we assessed the facial disability index and a clinical test registering the electromyographic activity, with and without biofeedback generated by Specular Face, a new software program; *Discussion:* After performing the appropriate tests, we checked the patients’ ability to change certain expressions when the Specular Face program was added. We can confirm that the mirror visual feedback therapy changes the behavior of synkinesis and the muscle function in these patients; *Conclusion:* The use of mirror therapy using a computerized treatment system of facial images yields promising results in modulating the muscle activity of patients with FP.

## 1. Introduction

Facial paralysis (FP) is a neuromuscular disorder caused by facial nerve injury [1]. From the motor standpoint, this facial nerve innervates the facial mimic muscles, the muscles of the stapes, stapedius, posterior belly of the digastricus and certain suprahyoid muscles. The facial nerve also collects sensory information from the external auditory canal and retroauricular region, as well as the gustatory and sensory nerve impulses of the anterior two-thirds of the tongue. The facial nerve is also responsible for collecting the parasympathetic innervation of the lacrimal gland and all salivary glands, with the exception of the parotid gland [2,3]. Among other clinical characteristics, facial nerve injury impedes the proper movement of various facial muscles and hinders facial expression, with dysfunction when closing the eyes, raising the eyebrows, smiling, talking and eating, among other actions [4].

Peripheral facial paralysis (PFP) entails lower motor neuron impairment [5,6], first described in 1829 by Charles Bell [7,8], and is the most common type of FP, accounting for approximately 60–75% of all cases of FP [9]. The etiology of PFP is uncertain, and although there are currently numerous etiopathogenic theories (vascular, viral and immunologic), none have been duly demonstrated [10,11]. There are conditions that can cause FP identical to PFP such as structural lesions in the ear or parotid gland, Guillain-Barre syndrome, Lyme disease, otitis media, Ramsay Hunt syndrome and sarcoidosis [5,12].

The physical manifestations of PFP include eyebrow sagging, inability to close the eyes, an upward and outward movement of the eye when trying to close the eyelids (Bell’s phenomenon), downturned mouth corners and loss of the nasolabial fold. There is also weakness or inability to perform movements with the orbicularis oris muscle [6,13]. Patients can also present xerostomia, ear pain (retroauricular), abnormal lacrimation and taste abnormalities [14].

In terms of the disease progression, a considerable percentage (50%) of patients with PFP have been reported to recover without the need for treatment; however, the main approach is medical [15]. Complementary to the medical treatment, physical therapy appears to be one of the treatments of choice in the process of recovering from PFP sequelae, with the most common treatments including physical therapy based on facial exercises, with or without feedback, in front of a mirror and manipulative therapy techniques [16]. The treatment is often directed towards the functional craniofacial problems associated with FP, and is therefore currently a symptomatic treatment.

New neuroscientific approaches for diseases that progress with chronic unilateral impairment have observed that mirror therapy (not in front of a mirror) could facilitate the postdisease reorganization of cortical neurons and help rebalance the intrahemispherical neuronal network, as well as improve motor function and participation in daily life [17]. Mirror therapy appears to work through the reactivation of the contralesional representation of the unreflected side of the body on the somatosensory cortex and motor cortex [17]. During mirror therapy, there is increased activity in the visual and somatosensory areas (primary and secondary), leading to better perception and increased application of attention resources, producing increased ipsilateral excitability while avoiding inhibition (interhemispherical) of the affected side when engaging in motor activation [18,19]. It has also been observed that mirror therapy activates the primary ipsilateral motor cortex, causing a beneficial effect in the affected hemisphere. This leads to the conclusion that mirror therapy is a promising, safe and effective tool for the functional recovery of patients with unilateral sensorimotor disorders [20,21].

We therefore present this case study as the first to report the electromyographic changes produced by using an “ad hoc” software program that generates a mirror image of an unaffected face for patients with FP. The objective was therefore to observe and describe the electromyographic gestural behavior in the orbicularis oris, buccinator and frontal muscles when adding facial mirror therapy using the new Specular Face software.

## 2. Procedure for the Cases

Patient recruitment was conducted through various physical therapy clinics and centers. We included patients who presented idiopathic PFP and FP secondary to surgery or disease. All patients were diagnosed by a neurologist specializing in neurophysiology or by an ear-nose-throat specialist. We also considered the presence of some type of associated disease. There was variability in the motor and sensory symptoms, which are described for each presented case. We also recorded the medications taken and the previous therapies prior to performing the study tests. The present study followed the international recommendations of the CARE checklist for drafting reports of case studies [22]. The study was approved by the ethics committee of La Paz University Hospital (HULP code PI-3228).

The study included the following instruments: (a) the Specular Face software, properly explained in the next paragraph; (b) an electromyography test to observe the gestural changes produced in the muscle activity of the orbicularis oris, buccinator and frontal muscles with the Specular Face software; and (c) the facial disability index (FDI) questionnaire to initially interpret the potential functional limitations and social participation of the study patients.

(a) The Specular Face Software: some of the authors of this article have developed the idea, concept and design of the Specular Face biofeedback. This software is designed to run on a laptop equipped with a camera, and starts by determining, in real time, whether there is a human face in the images it captures. If so, the software encloses the face within a rectangle and detects in it as many characteristic/feature points of a generic face (“corners” of eyes, nostrils, lips, etc.) as possible, using the method described by Saragih et al. [23]. It then builds a 2D triangular mesh with those feature points, and identifies its “vertical” symmetry axis, which might not be vertical in the image if the patient is tilting her/his head. Finally, Specular Face projects onto the laptop monitor, also in real time, composited images consisting of two halves: the “natural” one taken by the camera of the unaffected half of the face, plus a “synthetic” one which replaces the paralyzed half of the face by a mirrored version of the unaffected one. Specular Face was initially developed to run on macOS, and later extended to Ubuntu, arguably the most widespread Linux distribution. Its current version is written in C++, relies on OpenCV, the well-known open-source computer vision library, and runs seamlessly on a laptop with the following characteristics:

- CPU: Intel Core i7-6500U @ 2.5-3.1 GHz
- GPU: Intel HD 520
- RAM: 16 GB
- SSD: 512 GB
- Camera: 640 × 480 p @ 30 fps

(b) Electromyography: This technique helps study the muscle activation and provides information on the condition of the various motor unit components [24]. The electromyograph employed in this study is the FREEEMG (BTS Engineering Corp.), comprising variable geometry electrodes with snap connectors, a frequency of 1 kHz and a 16-bit resolution. The electrodes weigh 80 g, and the data is transmitted wirelessly. In the study, we employed three electrodes without changing their position between interventions (with or without feedback): one placed on the orbicularis oris, one on the buccinator and one on the frontal muscle. These electrodes capture the signal, which is then amplified, filtered and transformed into a digital signal. The electromyographic signal was treated using a band-pass filter, rectification and calculation of the root mean square during the temporally defined cycles for the requested gestures.

(c) Facial disability index questionnaire: FDI is a questionnaire whose objective is to assess the functional limitations and social participation of patients with FP. The index was designed by Van Swearingen and Brach in 1997, and was adapted to Spanish by Gonzales-Cardero in 2012. The questionnaire has high reliability for clinical practice and consists of 10 questions, five of which assess the physical function, while the other five assess the social function and wellbeing [25]. The following are the calculations described by the authors, and the maximum score for each of the parts is 100 [26]. The total is shown as the mean value of both parts divided by 100 (Table 1). The lower the score, the greater the associated disability.

## 3. Case Intervention

Initially, an appointment was made with each of the patients. The intervention consisted of a session in which the patients visited the measurements laboratory to undergo the corresponding evaluation. At the start of the appointment, the entire study procedure was explained, and the patients’ questions were answered. The patients were then asked to sign the informed consent document, and fill out a series of questions and data related to their paralysis, as well as the FDI questionnaire. The patients then went to a room with adequate lighting and temperature to prevent them from feeling cold, avoiding the possibility of increased spasticity. Patients sat on a chair with their feet on the floor, the knees and hip joints at a 90° angle and the seat back inclined by approximately 10°. One of the researchers then attached the wireless electrodes to the affected side of the orbicularis oris, buccinator and frontal muscles to record the activity of each muscle using the electromyograph. The first measurement was then taken to determine the activity of these three muscles without the use of mirror therapy or the software. Patients were asked to perform three gestures, always in the same order: a kiss, smile and raising of the eyebrows. These gestures had to be maintained by the patient for 5 s, after which they were given 10 s of rest between gestures. One of the researchers gave the first signal to the patients when starting the gesture of blowing a sustained kiss. The researcher then gave the signal to the patients to perform a sustained smile. Lastly, the patients raised their eyebrows for 5 s.

**Figure 1:**
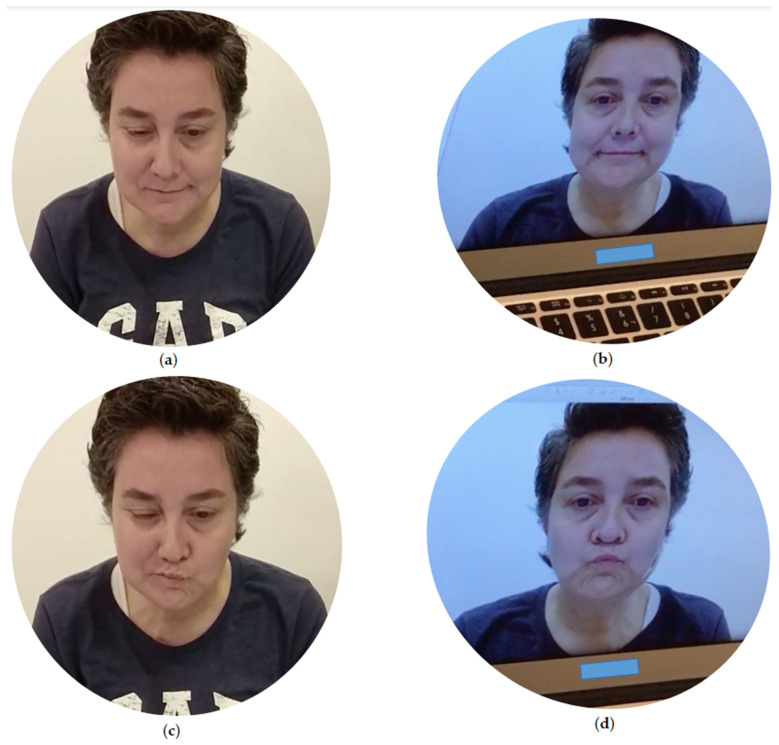
Representation of (**a**) real smile gesture; (**b**) simulated smile gesture by Specular Face at real time; (**c**) real kiss gesture and (**d**) simulated kiss gesture by Specular Face at real time.

A computer was then placed on the table, projecting the Specular Face program (in its beta version), which shows the healthy side of the patient’s face duplicated onto the affected side, thereby hiding the electrodes with this image. Once the mirror image was generated, the patients were asked once again to perform the previously executed exercises (kissing, smiling and raising of eyebrows), which were dynamically projected on the monitor in real time. Figure 1 shows the visual effect produced by Specular Face in performing the kissing and smiling gestures. In this protocol, the computer screen was set at a distance of 50–60 cm from the patients, with the top of the screen at eye height or slightly lower to generate a mirror image of the face that was as real as possible. Table 2 lists the numerical data corresponding to the millivolts of electromyography, represented as the original value ×10^3^.

## 4. Case Description

The description of each case includes the sex, age, profession, type of paralysis, noteworthy history and symptoms, both when the paralysis appeared and at the time of the study intervention. At the end of each case story, we included the FDI of each patient and described the observations in the electromyography of each gesture in the Supplemental Materials. The added gesture can be distinct in each case, given that we included the most visually relevant gesture, and where there was a greater change when introducing the Specular Face program. Based on previous studies, we considered clinical changes to be relevant starting at 20% [27]. Some electromyography graphs of the study cases and statistical data (including means, standard deviation and *d* Cohen size effect) are available in the Supplemental Materials.

### 4.1. Case 1

A 45-year-old businesswoman presented right FP secondary to a surgical procedure for acoustic neuroma two years earlier. The patient presented no systemic disease or associated disease.

Her FP has progressed for two years, and the symptoms are receding. However, the patient reports paresthesia and sensitivity exacerbation on the right side. She also reports a complete lack of saliva production and lacrimation on the right side. The patient presented complete deafness on the right side due to sectioning of the auditory nerve. She reported daily life difficulties in eating and drinking, as well as a complete loss of taste.

As treatment over the past two years, the patient has undergone acupuncture sessions once a week, with a one-month rest between each prescribed set of 10 sessions. At the start of her FP, the patient was referred to physical therapy sessions (last session, October 2017); she states that she wants to continue the rehabilitation. FDI score: 0.435.

### 4.2. Case 2

A 52-year-old female chemical engineer presented right PFP, which appeared on the 9th of October 2017 and was classified as highly severe. The patient presented no type of associated disease.

Since the start of her FP seven months earlier, her condition has improved. The patient stated that at the start of the paralysis she had difficulty eating, talking and closing the affected eye. This last difficulty resulted in the onset of keratitis, entailing difficulty watching television and using the computer. Currently, the main difficulties are when eating, which are caused by the onset of synkinesis. The patient also complains of pain on the right side of the face.

She is currently taking prednisone and applying Hialogel, Thealoz and VitA-POS for the eye. She has attended physical therapy sessions (massage therapy, electrotherapy and therapeutic exercise) and acupuncture sessions. FDI score: 0.375.

### 4.3. Case 3

A 46-year-old male teacher presented idiopathic FP on the side right of the face, which progresses without pain. The patient presented no systemic disease or associated disease.

The paralysis appeared six months ago as the result of a dental extraction. The FP had a severity of 5 out of 6 at the time of the diagnosis, which can be classified as highly severe. At the start, the patient took corticosteroids and anti-inflammatory agents. He is currently not taking any type of medication.

The patient is currently attending rehabilitation sessions where he undergoes daily manual physical therapy and electrotherapy. He is also undergoing acupuncture once a week, and underwent three sessions of osteopathy. FDI score: 0.325.

### 4.4. Case 4

A 23-year-old female statistician presented FP on the left side of the face, which started six months earlier and was secondary to surgery for an acoustic neuroma. The patient presented neurofibromatosis type 2 as an associated disease.

The main symptoms have progressed since the onset of the FP. The patient currently is unable to easily eat, accurately pronounce the letter “s” and talk fluidly, and experiences continuous dryness of her left eye.

As concomitant treatments, she performs various activities for four days a week, including physical, speech and occupational therapies. The treatments performed for her paralysis include massage, muscle retraining and therapeutic exercise. FDI score: 0.17.

### 4.5. Case 5

A 38-year-old female primary school teacher presented right FP caused by Guillain-Barré syndrome of unknown etiology. She started with paralysis on the left side in September 2017, with complete paralysis occurring within hours. On the third day, she was diagnosed with Guillain-Barre syndrome, the PFP remained on the right side, and the left side improved, leaving only a constant blinking in the eye.

Since the FP began, the patient presented hearing hypersensitivity, which has currently been resolved. The patient also reports hypersensitivity to light, and therefore has to wear sunglasses when going outside. She also complains of points of pain on the face. The patient also complained of difficulties brushing her teeth and drinking. Due to a lack of coordination, she has difficulties pronouncing letters when talking quickly. She was a smoker, but ceased because the paralysis made her unable to smoke.

She underwent treatment with immunoglobulins for 10 days. She underwent two sessions of physical therapy to improve mobility, reduce stiffness and modulate the tone through massage, exercises and dry needling. She also underwent occupational therapy sessions to improve coordination focused on her profession. FDI score: 0.31.

## 5. Discussion

The present study describes the effects of mirror therapy generated by an ad hoc computerized system, on muscle activity in a case series of patients with idiopathic and secondary PFP. The study serves as a starting point for generating new treatment hypotheses in this promising line of research and innovation. By observing the progression of the electromyographic activity in Figure 1 and in the Supplemental Materials, we can clearly verify the changes produced by the Specular Face biofeedback. In general, these changes tend to cause an activation of the muscle agonist and inhibition of activity in other muscles not associated with the movement. We can also see that the patients with various types of PFP have improved involuntary discriminatory capacity of the muscle activity. Nowadays, the phenomenon of agonist-antagonist muscle coactivation is discussed with respect to its consequences for movement mechanics (such as increasing joint apparent stiffness, facilitating faster movements and effects on action stability), implication for movement optimization, and involvement of different neurophysiological structures [28]. In the present study we have determined that there is an improvement in EMG when agonist muscles increase their contractile capacity, antagonists are inhibited and synergists provide support without generating synkinesis. Therefore, and depending on the gesture performed, it would be more advisable for synkinesis not to appear and for the EMG signal to be as divergent as possible in the activity of different muscles.

Focusing on case 1 (FP secondary to neurinoma of the acoustic nerve), we observe the onset of synkinesis of the buccinator, orbicularis and frontal muscles when performing the kiss gesture without the use of the Specular Face program, increasing the activity of the frontalis muscle at the end of the contraction. In comparison, when performing the gesture with the inclusion of the Specular Face program, we observe more uniform and balanced muscle activation, with better discrimination of the muscle contraction. In this same gesture, the electromyography shows substantial changes in the buccinator and frontal muscles, when the activity of these muscles is reduced 50% and 80%, respectively, when the gesture is executed with the Specular Face program. The patient presented synkinesis when performing the gesture without biofeedback, which could be due to the fact that the facial muscles decrease muscle spindles–a highly important fact, given that prolonged interruption of facial nerve function can lead to abnormal muscle function and reduced cortical reorganization [29]. Revisiting Figure 1, the use of facial mirror therapy using the Specular Face program could be the cause to explain the reduced synkinesis in the buccinator and frontal muscles during the kiss gesture.

In case 2 (severe idiopathic PFP), the electromyographic analysis showed clinically relevant changes when performing the gesture of raising the eyebrows using the Specular Face program, with a 69% reduction in orbicularis muscle activity. This change shows synkinesis inhibition of the orbicularis muscle in this gesture. As with case 1, prolonged interruption of facial nerve function can lead to abnormal function and impaired cortical reorganization [29]. Mirror therapy appears to facilitate cortical reorganization, help rebalance the intrahemispherical neuronal network and improve motor function [17]. This result could serve as an argument in favor of using mirror therapy with the Specular Face program for patients with some type of FP. In this same gesture, however, we observed no electromyographic changes in the frontalis muscle, which might be due to interference caused by cropping the mirror image created by the program, as well as the fact that, during the execution, part of the electrode located on the frontalis muscle was visible.

In case 3 (idiopathic FP), when performing the smile gesture with and without feedback, the activity of the frontal and orbicularis muscles maintained a continuous activity throughout the contraction; however, the activity of the buccinators was greater when the smile gesture was performed with feedback. In this gesture, the electromyography showed relevant changes in the buccinators muscle, with a 25% increase in muscle activation with the Specular Face program. This biofeedback could represent better somatosensory afferents produced by the visual stimulus, which would entail more efficient cortical activation and hence better muscle activation: more regular and uniform. Mirror therapy could also involve a reduction in muscle fatigue, due to the fact that it stimulates more coordinated synergistic contractions [30].

In case 4 (FP secondary to acoustic neuroma surgery), the gestures performed by the patient did not exceed a 20% change; therefore, there were no clinically relevant changes in this case. In the patient’s electromyograph, we can see that performing the gesture with feedback generated a more coordinated contraction of the orbicularis and buccinator muscles, maintained over the course of the gesture. The changes in muscle activity might be due to the fact that mirror therapy promotes fine motor recruitment and coordination between synergistic muscles involved in the gesture, which would entail decreased synkinesis [31]. It should be noted that this case of FP was secondary to surgery, and the damage to the facial nerve could largely explain the absence of clinically relevant differences.

In case 5 (right FP caused by Guillain-Barré syndrome), although we do not see greater changes in the smile gesture, the electromyography data shows clinically relevant changes in the kiss gesture. The electromyographic activity in the buccinator and frontal muscles is reduced by 25% when the kiss gesture is executed, which could be due to the patient’s underlying disease, in this case Guillain-Barre syndrome. The literature indicates that individuals with this type of disease can ultimately present disability in maintaining the strength of the muscle contraction over time. When motor conduction is affected, it entails a reduction in motor unit recruitment, which triggers an early onset of muscle fatigue [32,33]. This situation could explain the reduced activity during the smile gesture observed in this patient.

After analyzing each case individually, we checked the capacity for change when adding mirror therapy using the Specular Face program for certain gestures. In each case, the changes triggered when adding the program are interpreted differently. In general terms, we can confirm that mirror therapy causes changes in synkinesis and muscle function in these patients. The Specular Face program can also increase muscle agonist activity in patients with muscle weakness when performing a specific gesture. Additionally, there are relevant differences between the cases where the paralysis is secondary to surgery (cases 1 and 4), which could be explained by the changes in the progression time (increased progression time in case 1) and the results of the FDI (less disability in case 1). This clinical difference, which was greater in the patient with longer impairment and less disability, could be due to the generation of a mental adaptation of the facial image, when seeing that their paralyzed side was more similar to the unaffected side. There were also significant differences between cases of idiopathic facial paralysis (cases 2 and 3). In these cases, however, we cannot explain the cause of these differences, given that both cases of paralysis had similar progression times, equal severity and virtually identical FDIs. However, this conclusion requires further research with a broader sample, creating clinical trials specific for each type of paralysis, given the limited number of patients in this preliminary study.

In any case, the final development of the Specular Face should allow any patient to use it from their mobile device. However, given that the current Specular Face prototype occupies at times up to 90% of the PC’s Central Processing Unit, the mobile device that could run this “app” would have to be high-end and equipped, for example, with a Snapdragon 800 series chipset or similar, and of course, a latest generation mobile Graphics Processing Unit. Of the devices currently on the market, perhaps the Samsung Galaxy Tab S3 tablet or similar could be enough.

When performing the study, we encountered a number of limitations. First, the literature on this topic is scarce, and these are preliminary results supported by a very small group of patients. There are a small number of studies on mirror therapy with patients with FP (the most relevant were conducted by Ding et al. and Kang et al. [34,35]), which is a limitation due to the lack of protocols on how to conduct this therapy with patients with FP. Second, the results of this study should be taken with caution, as the patients’ consumption of drugs due to their medical treatment for other diseases – in those cases that had them – could have altered the reported results. Third, the patients included in the present study were not randomly selected. We performed a convenience sampling, which represents a limitation due to the inability to make general statements with statistical rigor. Fourth, we have the limitation of the software design, given that this currently involves a preliminary version that depends on the light levels in the room in which the measurements are performed. With the current software, the image clipping affected some of the patients when visualizing the eyebrow. There was also the loss of data when capturing the electromyography readings into numerical data. Furthermore, the EMG data reflect single trials for each case in each condition and differences between conditions would be more stable if more trials were run per condition, although due to the difficulty of transportation and time, excessive use of consumables and the fatigue caused by exposure to multiple repetitions (trials), it was decided that participants would only do two trials, the first of which was a practice and understanding trial of the experiment and the second of which was the recorded cycle. Lastly, future studies should improve on these limitations, focusing more specifically on the type of disease and adding the time of impairment, and performing clinical trials or observational studies.

## 6. Conclusions

Mirror therapy with the Specular Face program suggests electromyographic changes in patients with idiopathic FP secondary to surgery and secondary to Guillain-Barre syndrome. However, continued investigation and longitudinal studies with larger and more homogeneous patient groups (in terms of diagnosis and progression time) are needed.

The use of mirror therapy with a computerized treatment system of facial images is a promising approach for improving the quality of muscle activity, and inhibiting and reducing the synkinesis associated with voluntary facial movements in patients with PFP.

## Figures and Tables

**Table 1:** Descriptive data of Facial Disability Index.

FDI	PHYSICAL (0–100)	SOCIAL (0–100)	TOTAL (0–1)
Case 1	35	52	0.44
Case 2	35	40	0.38
Case 3	65	0	0.33
Case 4	30	4	0.17
Case 5	50	12	0.31

FDI: Facial Disability Index.

**Table 2:** Maximum electromyographic data (millivolts) in patients with facial palsy.

Gesture	KISS						SMILE						EYEBROWNS					
Muscle	Orbicularis		Buccinator		Frontalis		Orbicularis		Buccinator		Frontalis		Orbicularis		Buccinator		Frontalis	
Bio-Feedback	WithO	With	WithO	With	WithO	With	WithO	With	WithO	With	WithO	With	WithO	With	WithO	With	WithO	With
Case 1	42	40	29	14	20	4	18	19	9	11	-	-	-	-	-	-	-	-
Case 2	10	33	7	8	-	-	32	10	8	8	-	-	32	10	-	-	57	64
Case 3	12	11	12	12	3	3	6	8	23	22	4	5	5	7	10	18	5	5
Case 4	5	5	4	5	2	2	3	3	5	4	3	3	4	4	2	2	4	3
Case 5	23	21	7	5	7	5	14	12	11	9	6	7	13	10	9	8	6	5

WithO, without Specular Face software; With, with Specular Face software. All data is expressed in millivolts (mV).

## Data Availability

The data presented in this study are available on request from the corresponding author. The data are not publicly available due to data protection reason.

## Supplementary Materials

The following are available online at https://www.mdpi.com/article/10.3390/brainsci11070930/s1, Supplemental Material: EMG graphics and analysis data for each case.
