# Supplementary material for: Influence of Mirror Therapy (Specular Face Software) on Electromyographic Behavior of the Facial Muscles for Facial Palsy
Source: Brain Sci. 2021 Jul 14;11(7):930. doi: 10.3390/brainsci11070930 (PMC8308022; doi:10.3390/brainsci11070930)

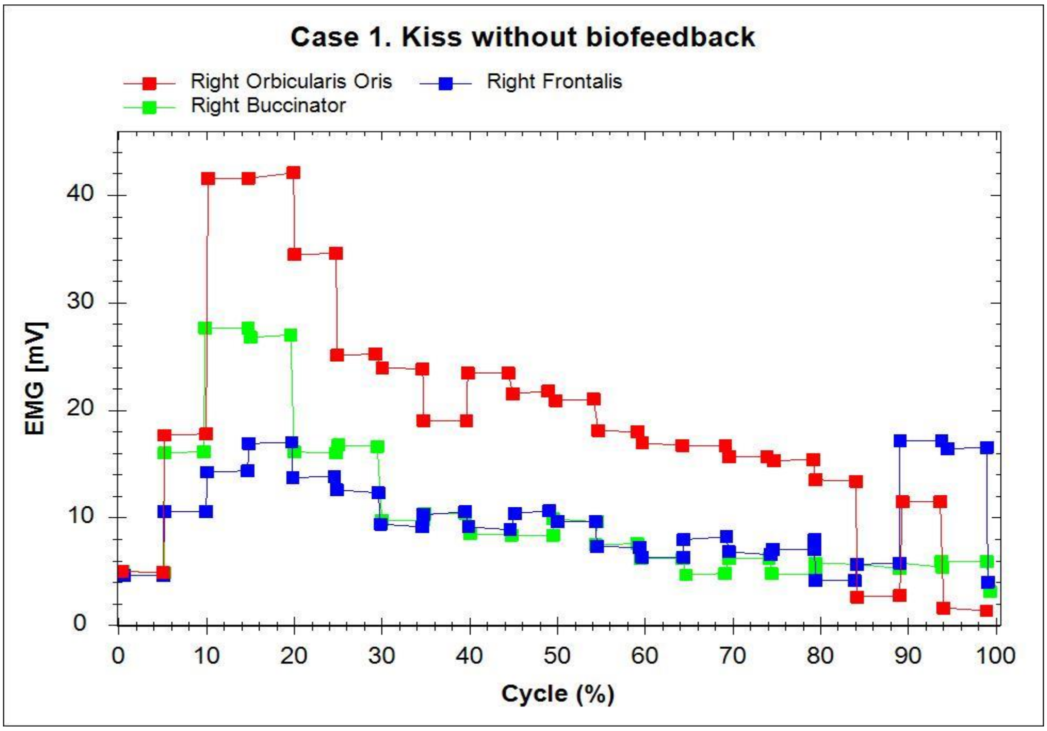

Muscle comparison of the mean muscle activity with and without feedback. Data are presented in means ± standard deviation and d Cohen size effect.

Muscle Pair	means ± standard (without-with feedback)	*d* Cohen
Orbicularis Oris (mV)	19.08 ± 10.5	-0.36*
	21.46 ± 7.47	
Buccinator (mV)	9.73 ± 6.15	-0.21
	10.82 ± 1.81	
Frontalis (mV)	9.45 ± 3.33	2.19**
	1.91 ± 0.91	

*p<0.05; **p<0.01

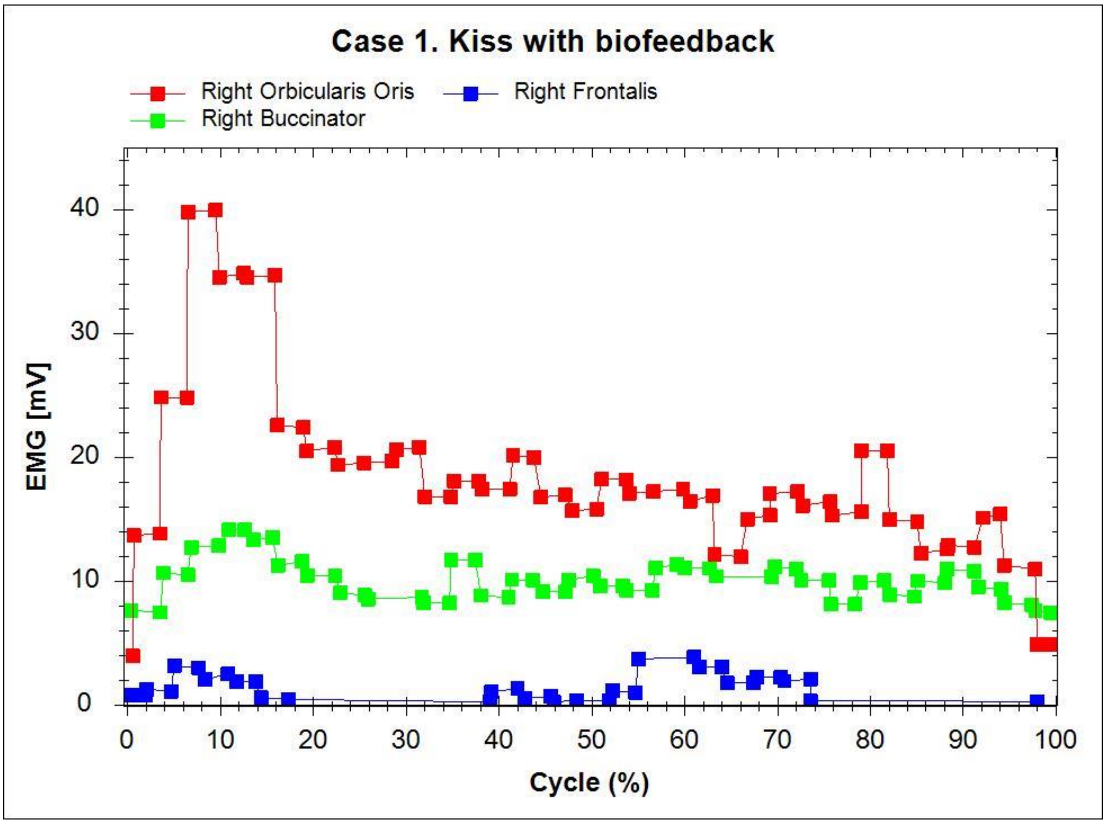

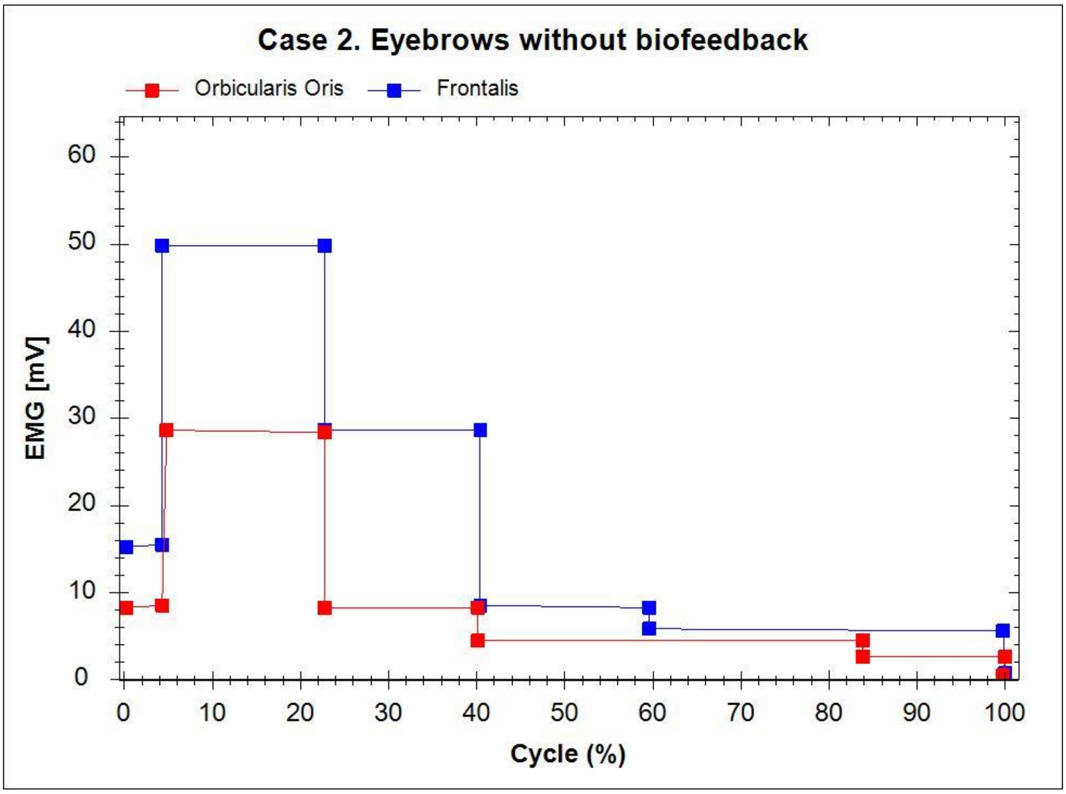

Muscle comparison of the mean muscle activity with and without feedback. Data are presented in means ± standard deviation and d Cohen size effect.

<table>
<tr><th>Muscle Pair</th><th>means ± standard (without-with feedback)</th><th>d Cohen</th></tr>
<tr><td rowspan="2">Frontalis (mV)</td><td>19.91 ± 17.18</td><td rowspan="2">-1.22**</td></tr>
<tr><td>34.55 ± 21.72</td></tr>
<tr><td rowspan="2">Orbicularis Oris (mV)</td><td>10 ± 9.79</td><td rowspan="2">0.24</td></tr>
<tr><td>7.91 ± 2.81</td></tr>
</table>

*p<0.05; **p<0.01

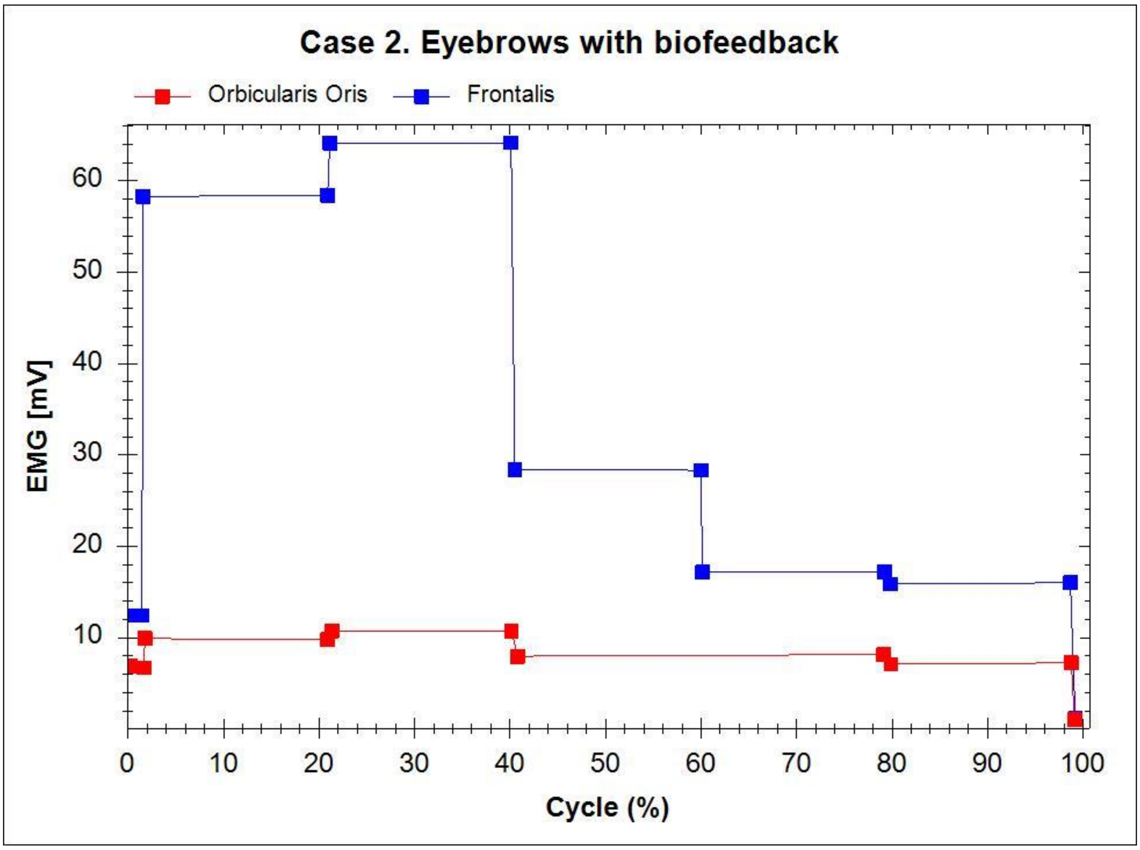

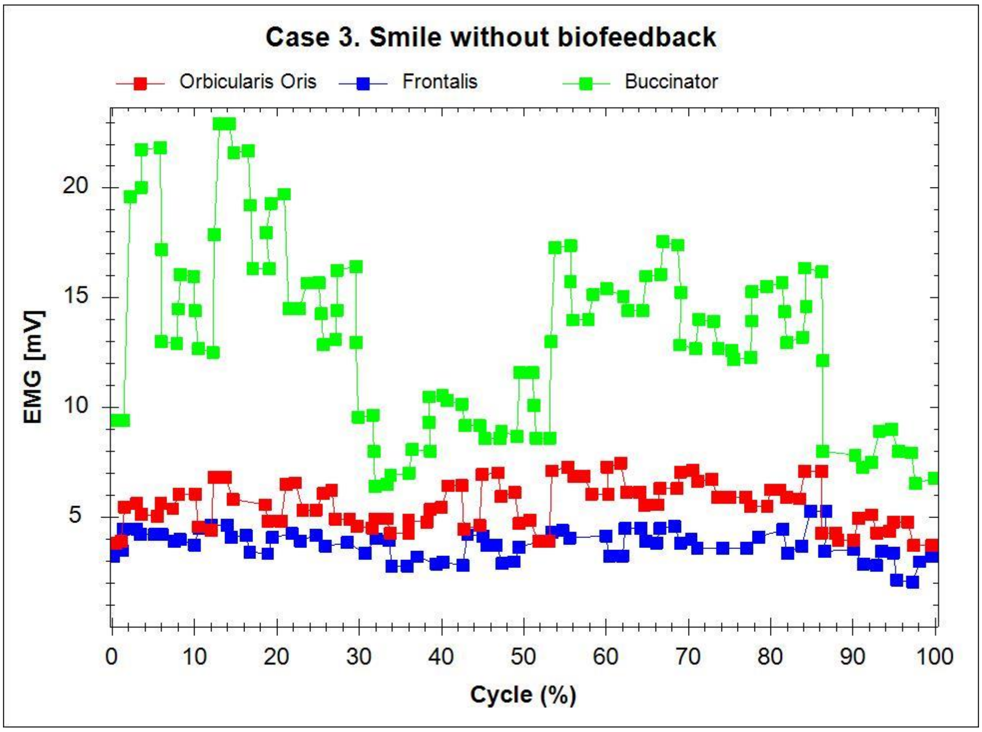

Muscle comparison of the mean muscle activity with and without feedback. Data are presented in means ± standard deviation and d Cohen size effect.

Muscle Pair	means ± standard (without-with feedback)	*d* Cohen
Buccinator (mV)	13.64 ± 4.44	-0.23*
	14.77 ± 3.52	
Orbicularis Oris (mV)	5.61 ± 0.9	0.34**
	5.21 ± 0.94	
Frontalis (mV)	3.95 ± 0.49	0.53**
	3.49 ± 0.72	

*p<0.05; **p<0.01

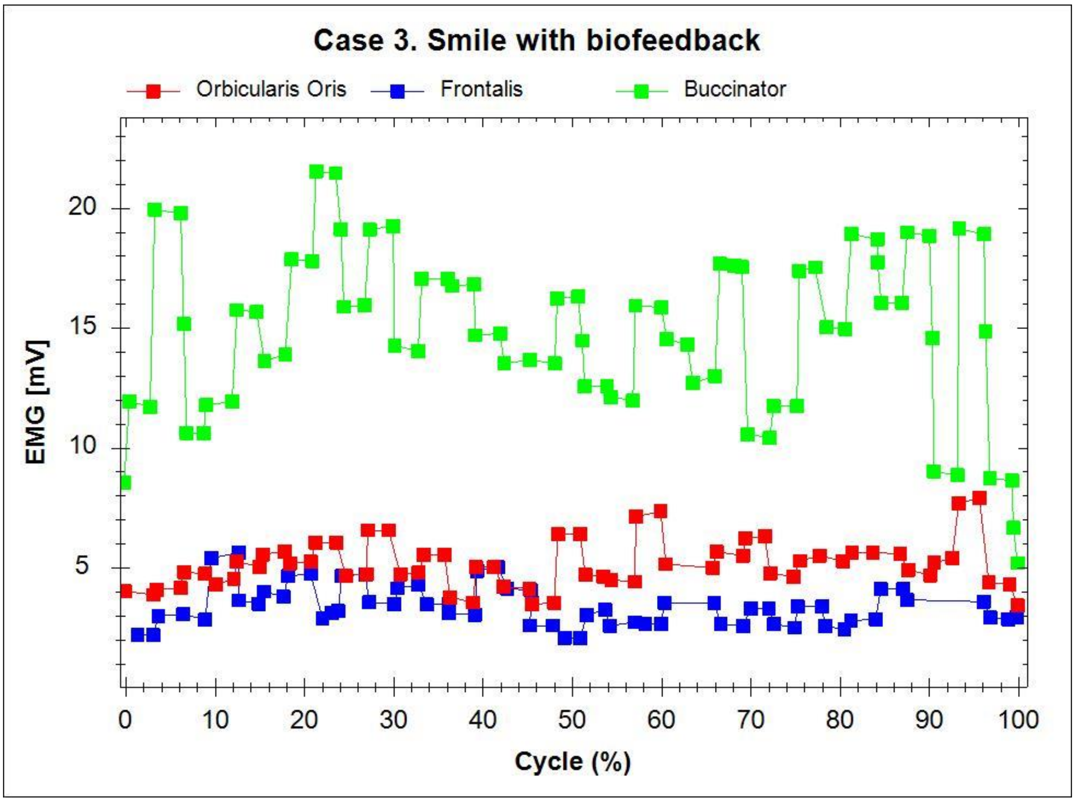

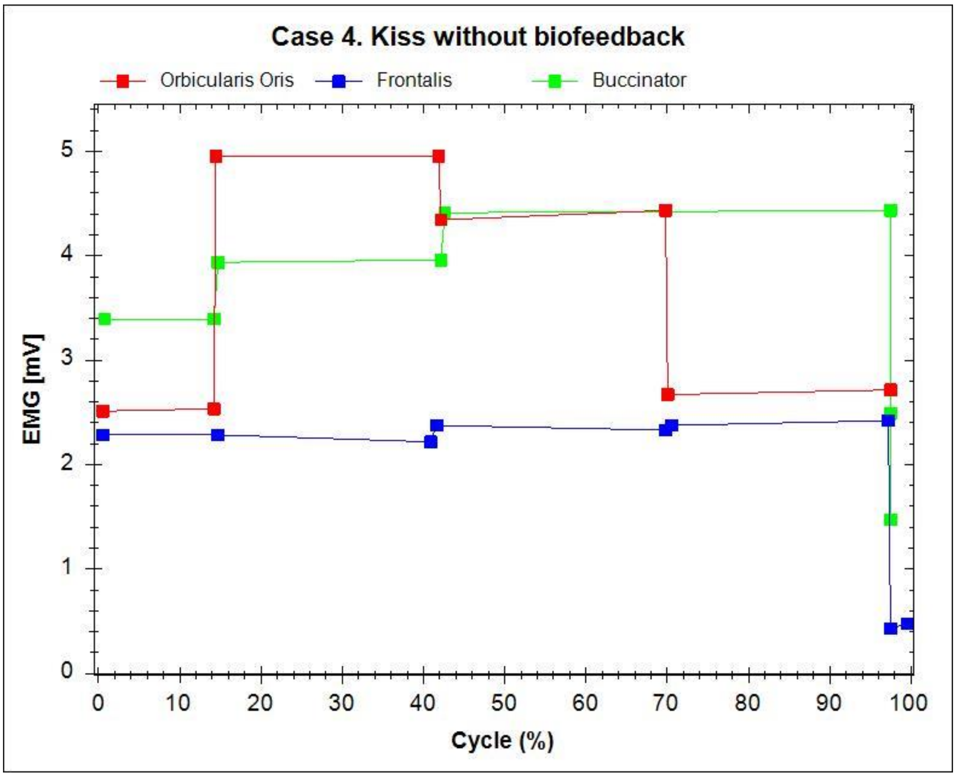

Muscle comparison of the mean muscle activity with and without feedback. Data are presented in means ± standard deviation and d Cohen size effect.

Muscle Pair	means ± standard (without-with feedback)	*d* Cohen
Orbicularis Oris (mV)	3.65 ± 1.15	-0.26
	4.03 ± 1.02	
Buccinator (mV)	3.53 ± 0.98	-0.16
	3.71 ± 0.29	
Frontalis (mV)	2.09 ± 0.64	-0.94*
	2.73 ± 0.09	

*p<0.05; **p<0.01

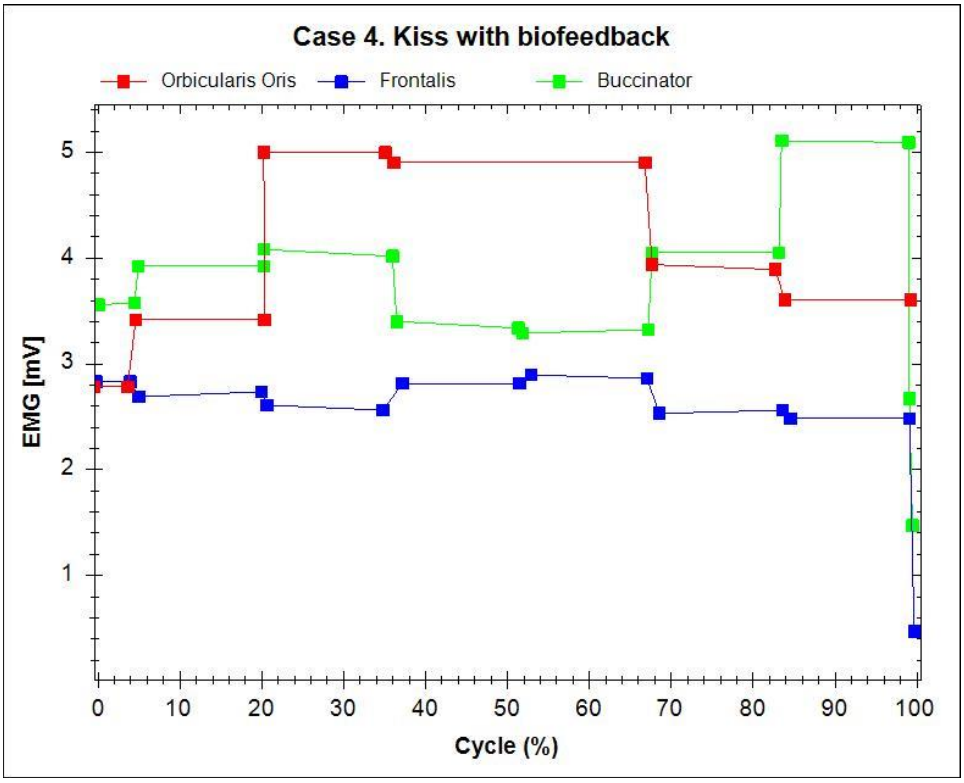

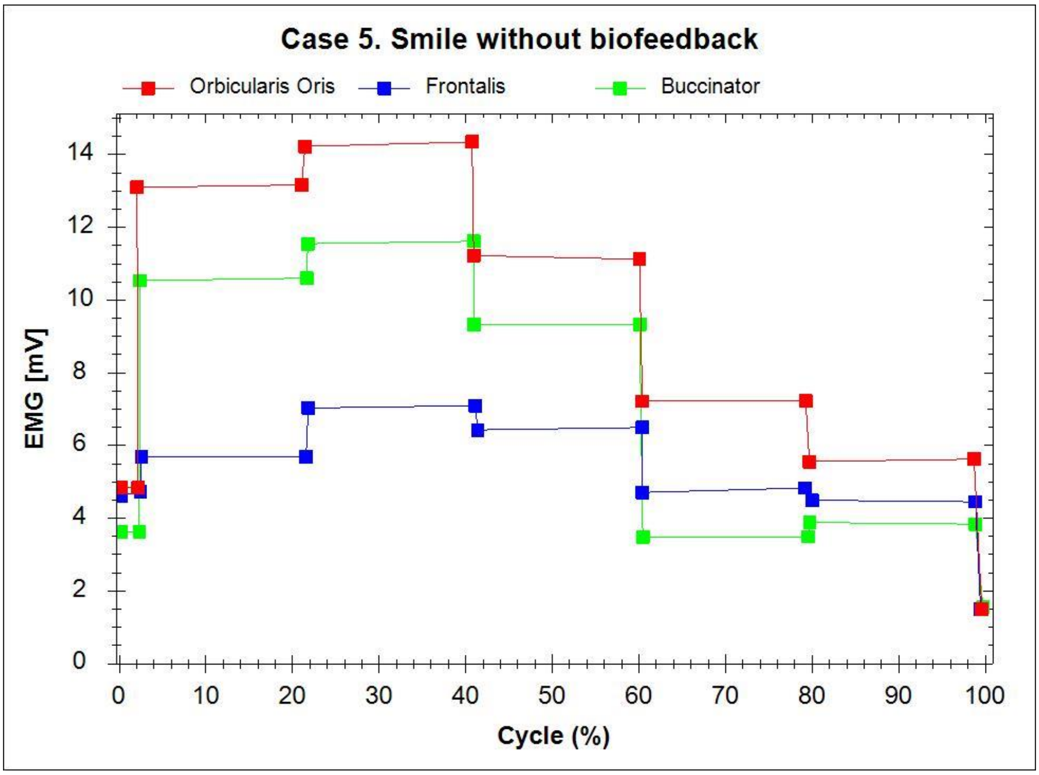

Muscle comparison of the mean muscle activity with and without feedback. Data are presented in means ± standard deviation and d Cohen size effect.

Muscle Pair	means ± standard (without-with feedback)	*d* Cohen
Buccinator (mV)	7.02 ± 3.49	0.28
	6.51 ± 2.63	
Orbicularis Oris (mV)	8.75 ± 4.19	0.27
	8.35 ± 3.05	
Frontalis (mV)	5.62 ± 1	0.19
	5.36 ± 1.87	

*p<0.05; **p<0.01

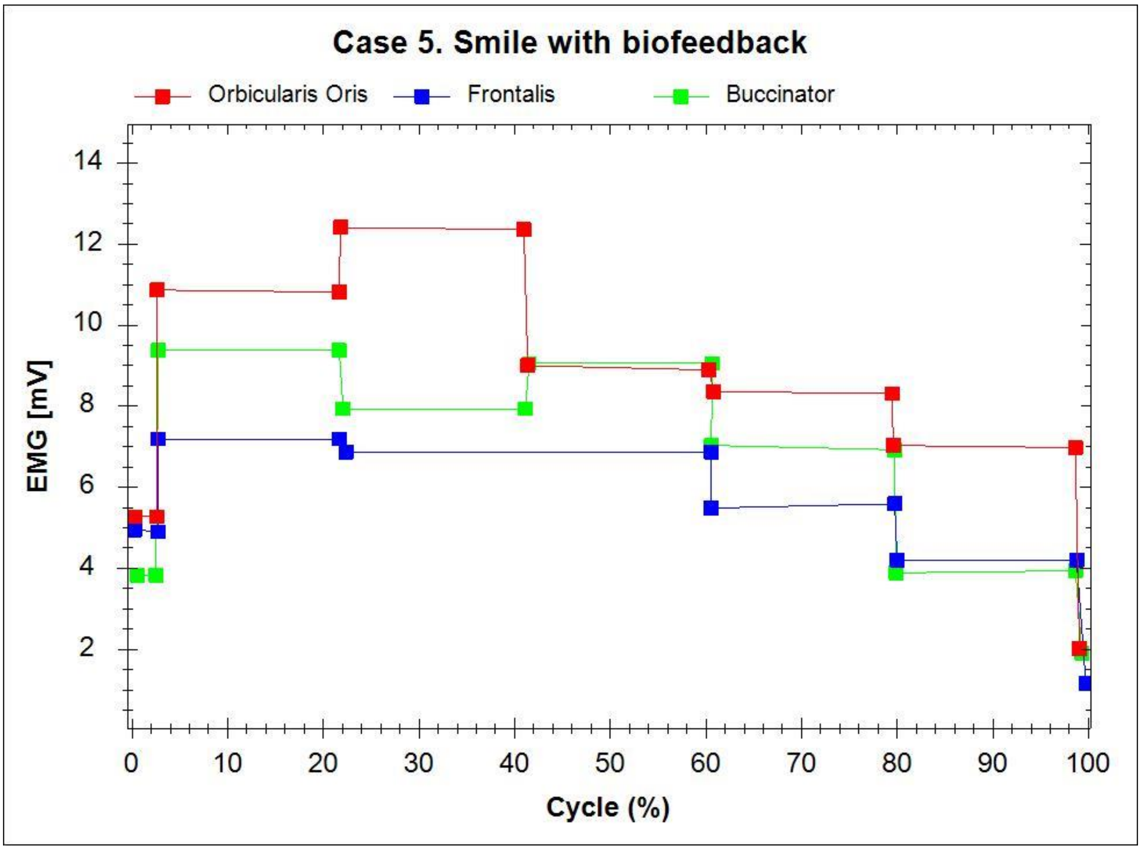

Supplement: Supplementary file 1 [file brainsci-11-00930-s001.zip › brainsci-1279699-supplementary.pdf]
